# Effects of Soil Warming and Nitrogen Addition on Soil Respiration in a New Zealand Tussock Grassland

**DOI:** 10.1371/journal.pone.0091204

**Published:** 2014-03-12

**Authors:** Scott L. Graham, John E. Hunt, Peter Millard, Tony McSeveny, Jason M. Tylianakis, David Whitehead

**Affiliations:** 1 School of Biological Sciences, University of Canterbury, Christchurch, New Zealand; 2 Landcare Research, Lincoln, New Zealand; 3 Department of Life Sciences, Imperial College London, Silwood Park Campus, Ascot, Berkshire, United Kingdom; University of Maryland, United States of America

## Abstract

Soil respiration (*R*_S_) represents a large terrestrial source of CO_2_ to the atmosphere. Global change drivers such as climate warming and nitrogen deposition are expected to alter the terrestrial carbon cycle with likely consequences for *R*_S_ and its components, autotrophic (*R*_A_) and heterotrophic respiration (*R*_H_). Here we investigate the impacts of a 3°C soil warming treatment and a 50 kg ha^−1^ y^−1^ nitrogen addition treatment on *R*_S_, *R*_H_ and their respective seasonal temperature responses in an experimental tussock grassland. Average respiration in untreated soils was 0.96±0.09 μmol m^−2^ s^−1^ over the course of the experiment. Soil warming and nitrogen addition increased *R*_S_ by 41% and 12% respectively. These treatment effects were additive under combined warming and nitrogen addition. Warming increased *R*_H_ by 37% while nitrogen addition had no effect. Warming and nitrogen addition affected the seasonal temperature response of *R*_S_ by increasing the basal rate of respiration (*R*_10_) by 14% and 20% respectively. There was no significant interaction between treatments for *R*_10_. The treatments had no impact on activation energy (*E*_0_). The seasonal temperature response of *R*_H_ was not affected by either warming or nitrogen addition. These results suggest that the additional CO_2_ emissions from New Zealand tussock grassland soils as a result of warming-enhanced *R*_S_ constitute a potential positive feedback to rising atmospheric CO_2_ concentration.

## Introduction

Soils contain a pool of carbon approximately double that stored in terrestrial biomass [1]. Soil respiration (*R*_S_), the primary pathway for return of soil carbon to the atmosphere, is increasing globally by 0.1 Pg C y^−1^ at present [2]. This increase, hypothesised to be a result of global warming, is concerning as temperatures are expected to rise by as much as 6.4°C over the next century [3]. Coupled climate models indicate a likely soil-driven positive feedback to climate change, although uncertainty remains in the magnitude of this feedback [4], [5].

Numerous warming experiments have investigated the impacts of long-term climate warming on carbon cycling, suggesting that, on average, warming of 0.3–6.0°C increases soil respiration (*R*_S_) by 20% [6]. However, several notable examples have shown the effect of warming on *R*_S_ to be only transient [7], [8]. Mechanisms for this acclimation of *R*_S_ to prolonged warming include depletion of labile carbon substrates [8]–[10], changes to the microbial community structure [7], [11], physiological acclimation of soil microbes [12], reduction in root biomass [13] and reduction in the specific root respiration rate [14]. Acclimation of soil respiration may limit potential soil carbon loss as a result of climate warming.

Global change scenarios also suggest that nitrogen cycling in terrestrial ecosystems will be altered. Nitrogen deposition due to crop fertilisation and fossil fuel combustion currently exceeds terrestrial nitrogen fixation and is expected to increase in the future [15]. As warming also increases nitrogen mineralization [6], there exists the possibility for synergistic effects of warming and anthropogenic nitrogen deposition on plant-available nitrogen.

While warming-induced increases in *R*_S_ represent a likely positive feedback to rising atmospheric CO_2_ concentration, enhanced nitrogen deposition has been suggested as a possible mitigating factor due to negative impacts of nitrogen addition on *R*_S_ [16]. The findings from nitrogen addition experiments in forests suggest that reduction in *R*_S_ may represent a carbon offset equivalent to the nitrogen fertilisation effect on primary production. As well, reductions in *R*_S_ have been observed in grasslands as a result of nitrogen addition [17], [18]. Due to feedbacks between the nitrogen and carbon cycles, nitrogen availability will likely influence the magnitude of the terrestrial feedback to rising atmospheric CO_2_ concentration [19].

The net response of *R*_S_ to warming and nitrogen addition depends largely on the combined response of its components, autotrophic soil respiration (*R*_A_) and heterotrophic soil respiration (*R*_H_), which are likely to have different responses to environmental drivers. Autotrophic respiration refers to respiratory activity of roots and associated rhizosphere microbes, while *R*_H_ refers to soil organic matter decomposition by soil microbes [20]. The important distinction between *R*_A_ and *R*_H_ is that the former represents respiration of carbon recently assimilated by plants, whereas the latter releases carbon that may have residence times in the soil reaching millennia [21].

Heterotrophic respiration is widely expected to increase under warming scenarios [22], [23]. Several field warming experiments demonstrated such increases [24], [25]. While warming generally increases *R*_H_, nitrogen addition can decrease microbial biomass [26], potentially reducing *R*_H_. This reduction in *R*_H_ may explain the overall decrease in *R*_S_ observed in response to nitrogen addition [16].

In this study, we investigated the impacts of soil warming and nitrogen addition, as well as their interaction, on *R*_S_ and its components, *R*_A_ and *R*_H_. Such multifactor experiments are important to improve the predictive ability of coupled-climate models, as single factor experiments may fail to predict interactive effects of global change drivers [27], [28]. Likewise, partitioning the autotrophic and heterotrophic components of *R*_S_ can lead to greater mechanistic understanding of the response of *R*_S_ to environmental drivers [29].

Native tussock grassland was selected as a model system because grasslands are a widespread and important store of carbon in New Zealand [30], and globally [31]. Soil respiration and *R*_H_ were measured over a period of 27 months with the objective of determining the likely feedback effect that increases in *R*_S_ in grasslands will have on rising atmospheric CO_2_ concentration in response to soil warming and nitrogen addition.

## Methods

### Study site

This study was conducted at the Cass Warming Experiment at the University of Canterbury Cass Field Station in central South Island, New Zealand (43.03° S, 171.75° E, 590 m a.s.l.). The site was constructed in January 2009 in an area of tussock grassland. Soils at the site are classified as acidic allophane brown by New Zealand Soil Classification (Typic Dystrochrept by USDA) [32], [33]. Prior to this study, vegetation and the top 200 mm of topsoil were removed, twenty 12.25 m^2^ plots were laid out and 90 m of resistance heating cable (Argus Heating, Ltd., Christchurch, New Zealand) were arranged in rows with 140 mm spacing between cables in each plot to achieve a heating density of 76 W m^−2^ [34]. Dummy cables were arranged similarly in unheated plots. The cables were then covered with 200 mm of topsoil and the native New Zealand tussock grasses *Festuca novae-zelandiae* (50 individuals per plot), *Poa cita* (50 per plot), *Chionochloa rigida* (22 per plot), and *Chionochloa flavescens* (12 per plot) were planted.

Five plots were assigned to each of four treatments: control, warming only, nitrogen addition only and combined warming and nitrogen addition (Appendix S1). In each of 10 plots designated for warming, three thermocouples (Type-E, Campbell Scientific, Logan, UT, USA) were buried to a depth of 100 mm in a stratified design which captured a range of horizontal distances from heating cables (directly above, one quarter of the distance between two cables and the midpoint). In each of the control plots, one thermocouple was buried to 100 mm soil depth. The heating cables were switched on and off to maintain a 3°C difference between the average of the three thermocouples in warmed plots and the nearest un-warmed plot. Warming was controlled by a datalogger (CR1000X, Campbell Scientific, Logan, UT, USA) and hourly average plot soil temperatures were recorded. An auxiliary weather station measured hourly average air temperature, soil temperature and volumetric water content at 100 mm depth.

Nitrogen addition began in February 2009. Nitrogen was applied as calcium ammonium nitrate at a rate of 10 kg N ha^−1^ five times throughout the growing season to achieve a total amendment of 50 kg N ha^−1^ y^−1^. For each plot, the fertiliser was dissolved in 4 L water and distributed using a watering can over both plants and soil. The continuous 3°C warming treatment was started in July 2009. Two plots, one each of the warming only and combined warming and nitrogen addition treatments, were subsequently dropped from analyses due to malfunction of the heating cables.

### Respiration measurements

Measurements of soil respiration were carried out over a 27 month period beginning in August 2009 (winter) and continuing through October 2011 (spring). Six 100 mm diameter polyvinyl chloride measurement collars were installed to a soil depth of 70 mm in each plot. The rate of soil respiration in each collar was measured at 2–4 week intervals using a portable respiration system (SRC-1 and EGM-4, PP Systems, Amesbury, MA, USA). An additional two measurement collars were installed in each plot to a soil depth of 300 mm in order to exclude roots and provide an estimate of heterotrophic respiration (*R*_H_). These deep collars extended into the clay subsoil, limiting potential root growth into the soil beneath the collar. In contrast, the shallow collars were inserted to a depth that would allow root infiltration beneath the collar while providing a seal with the soil surface. The collars remained in place for the duration of the experiment to avoid soil or root disturbance. Measurement using the deep collars began in January 2010. Simultaneous with each soil respiration measurement, soil temperature and soil water content at 50 mm depth were measured using a thermocouple (Type-E, Omega Engineering, Ltd, Stamford, CT, USA) and a soil moisture sensor (Theta Probe type ML1 and ML2, DeltaT Devices, Cambridge, UK), respectively.

### Substrate addition

Availability of labile substrates in the soil is important in regulating *R*_S_ [35]. In order to assess levels of substrate limitation induced by warming and nitrogen addition treatments and the presence of roots, a substrate addition experiment was carried out in late-October 2011. In each of 16 plots representing four replicates for each treatment, two pairs of soil respiration measurement collars were selected: one pair with roots present and another pair with roots excluded. One collar from each pair was selected randomly for substrate addition. All collars were measured immediately prior to substrate addition. Subsequent to initial measurement, the two collars from each plot selected for substrate addition were amended with 20 ml of 0.2 M sucrose solution (an amount approximately equivalent to 10 days of carbon losses from *R*_H_). In order to ensure that the sucrose solution infiltrated beyond the soil surface, 5 ml were injected with a syringe to a depth of 25 mm at four locations within each collar. The collars designated as controls were treated similarly with water. Soil respiration was then measured in each collar at 30 min, 1 h, 2.5 h, 4 h and then at 4–8 h intervals until the substrate response was no longer evident. Substrate-induced respiration (*S*_I_) was calculated for each pair of collars as the difference between respiration rates of the control and substrate-added collars, as a proportion of the rate for the control treatment.

### Soil analyses

Soils were sampled in January 2010, March 2011 and March 2012. Three 54 mm diameter soil cores were taken to a depth of 100 mm in each plot and the soil was homogenized into a single sample. Roots were removed by 8 mm sieve and dried at 60°C. As the grass roots were very fine, in 2012 a subsample was removed from the whole sample and washed over a 650 μm sieve to obtain root biomass. Microbial biomass was estimated using the fumigation-extraction technique adapted from Vance et al. [36]. Remaining soil was dried at 60°C, passed through a 2 mm sieve to remove remaining roots and ground in a ball mill. Samples were then analysed for total carbon and nitrogen concentration on an elemental analyser (CNS2000, Leco, St. Joseph, MO, USA).

Between 20 September 2011 and 22 October 2011, plant available nitrogen was estimated using ion exchange membranes (PRS probes, Western Ag Innovations, Saskatoon, Canada). The PRS probes were installed to a depth of 100 mm at three locations in each plot. Following a one-month burial period, probes were removed, rinsed with deionized water and returned to Western Ag Innovations for analysis of NH_4_^+^ and NO_3_^−^.

### Statistical analyses

The effects of warming and nitrogen addition on seasonal measurements of *R*_S_, *R*_H_ and soil water content were assessed using linear mixed-effects models conducted in the ‘nlme’ package [37] in R version 2.12.1 [38]. Warming, nitrogen addition and measurement date, along with their interactions were included as fixed effects, while measurement collars nested within plots were included as random effects to account for the non-independence of multiple samples through time and multiple collars per plot. Residual analyses were undertaken and log transformation was used for *R*_S_ and *R*_H_, to correct for heteroscedasticity. The effects of warming and nitrogen on the proportion of *R*_S_ constituted by *R*_H_ (*f*_RH_) were similarly assessed by treating plot averages of *f*_RH_ on a given date as a sample and evaluating random effects at the plot level.

Temperature responses of *R*_S_ and *R*_H_ were fitted to an Arrhenius-type curve [39], modified with a soil water content response function [40]:
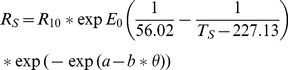
(1)where *T*_S_ is soil temperature (K), *R*_10_ is the basal respiration rate at 10°C, *E*_0_ is the activation energy of enzymatic reactions, *θ* is the soil volumetric water content and *a* and *b* are parameters that determine the shape of a sigmoidal response of respiration to soil water content.

Nonlinear mixed-effects models (also conducted in the ‘nlme’ package for R) were used to fit Equation (1) initially to measurements of *R*_S_, and subsequently to *R*_H_. First, the effect of roots on the temperature response of *R*_S_ was investigated by testing how root presence (as a fixed effect) altered parameter values for *R*_10_ and *E*_0_. Subsequently, temperature responses of *R*_S_ and *R*_H_ were investigated in separate models, with the latter substituting *R*_H_ in place of *R*_S_ in Equation (1). Warming and nitrogen addition, as well as their interactions, were investigated as fixed effects on *R*_10_ and *E*_0_ for both the *R*_S_ and *R*_H_ models. For all the above nonlinear models, measurement collars nested within plots were evaluated as random effects. Fixed and random effects on *a* and *b* were not evaluated, as few measurement dates occurred under water-limited conditions, and a generic water content response curve which limited respiration when soil water content was less than 0.2 m^3^m^−3^ was deemed appropriate based on analysis of residuals of a temperature-only model.

The final fixed effects structure was determined by first constructing a maximal model which included the presence of plant roots, warming, nitrogen and all interactions. A power variance function was fitted in order to correct for heteroscedasticity [41]. To account for autocorrelation in repeated measurements of the same collar, a first order autoregressive structure was used [42]. Fixed effects and interactions were removed iteratively based initially on their p-values and the best fit model was selected. During each step of this procedure, model comparisons were undertaken using a likelihood ratio test and selection of the best-fitting model was achieved through minimisation of Akaikie's Information Criterion (AIC). To test for potential acclimation of *R*_S_ to warming, the data were bisected such that the first full year of measurement was separated from the second. The interaction between measurement year and the warming treatment was added as a fixed effect on both *R*_10_ and *E*_0_ parameters. This interaction was tested for significance to determine whether the warming effect was consistent across measurement years, with a significant negative interaction term for either parameter indicating acclimation.

Soil carbon content, soil nitrogen content, microbial biomass, plant available nitrogen and substrate induced respiration (*S*_I_) were all assessed by multi-way ANOVA, with temperature and nitrogen treatments, as well as their interaction, as factors. For *S*_I_, the maximum value recorded for each pair off collars over the measurement period was tested. For those variables that were measured repeatedly (soil carbon, soil nitrogen, microbial biomass carbon), a separate ANOVA was conducted for each time point.

## Results

### Seasonal variation of soil temperature and water content

**Figure 1:**
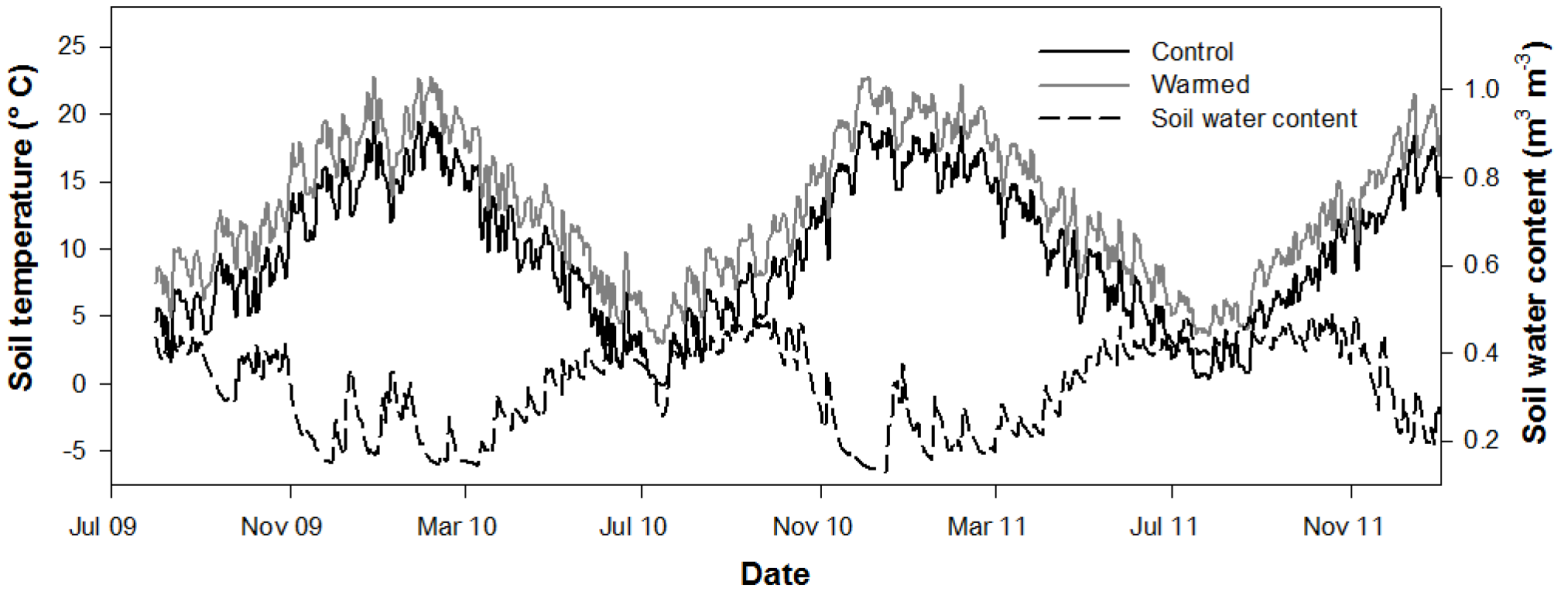
Seasonal variations in soil variables. Measured soil temperature, *T*_S_ (°C), and soil water content, *θ*, at 100 mm depth.

Average soil temperature over the entire 27 month measurement period was 9.6°C. Warming increased soil temperature by an average (±SE) of 3.1±0.2°C over the course of the experiment (Fig. 1). Soil water content varied seasonally, falling below 0.2 m^3^ m^−3^ periodically during summer and remaining above 0.40 m^3^ m^−3^ during the winter months. Warming significantly reduced soil volumetric water content (p = 0.001, Table S1) by an average 0.01 m^3^ m^−3^. This reduction in soil water content was most evident in summer when water was limiting, with maximum reduction in soil water content of 0.04 m^3^ m^−3^ observed in February 2010 and 0.07 m^3^ m^−3^ in January 2011.

### Seasonality of R_S_

**Figure 2:**
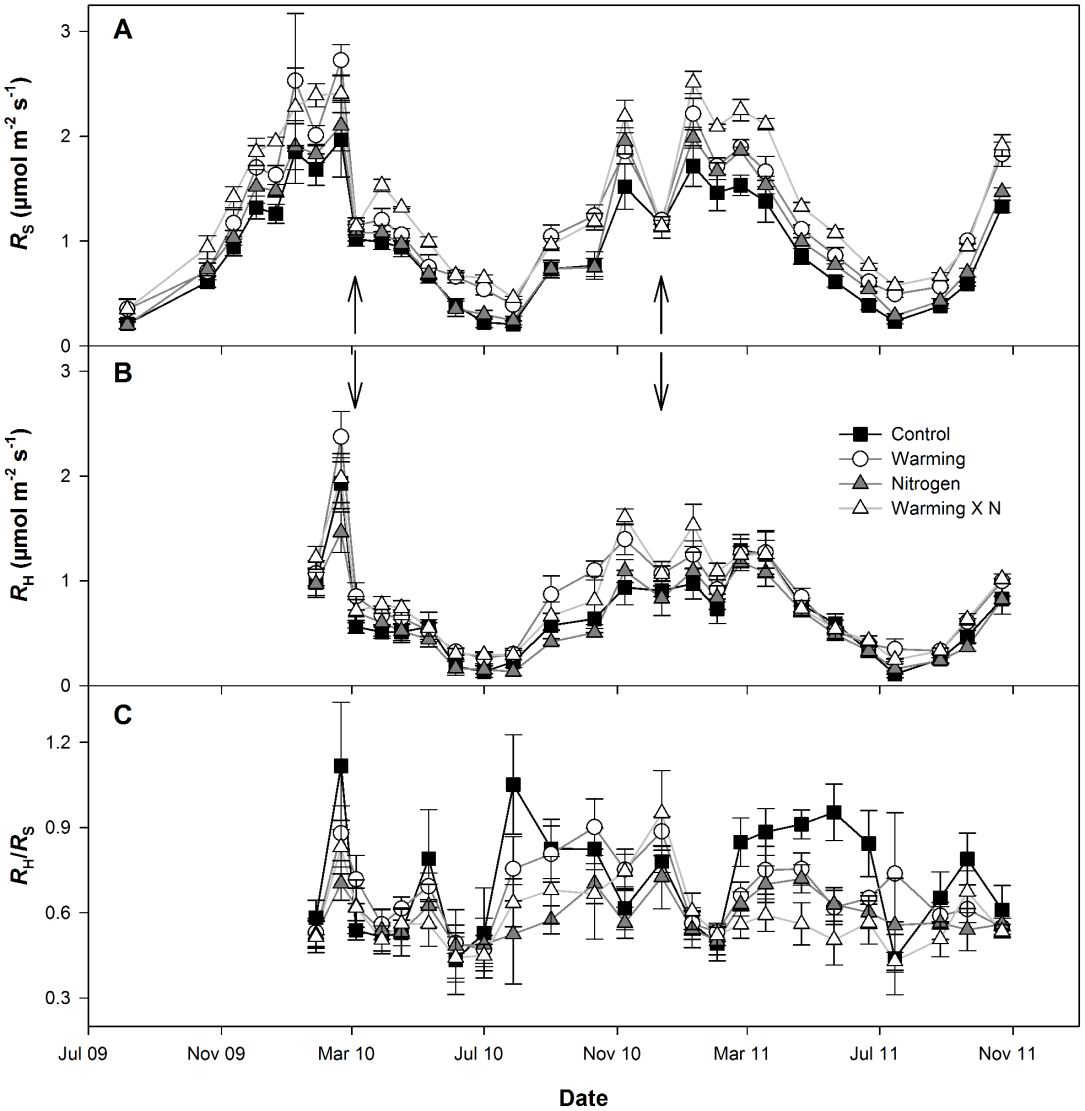
Seasonal rate of soil respiration. Mean ± SE soil respiration *R*_S_ (A), hetrotrophic respiration, *R*_H_ (B), and the proportion of total R_S_ contributed by *R*_H_ (*f*_RH_) (C) by date; arrows indicate dates for which soil water content was below 0.2 m^3^ m^−3^.

Soil respiration showed a seasonal pattern driven primarily by seasonal temperature (Fig. 1, Fig. 2A). As such, measurement date had a significant effect in the linear mixed-effects model (p<0.0001, Table S2). Heterotrophic respiration showed a similar response to seasonal temperature and, as such, measurement date was significant in predicting *R*_H_ (p<0.0001, , Fig. 2B). The average proportion of *R*_S_ constituted by *R*_H_ (i.e., *f*_RH_) was 71% (Fig. 2C). No apparent seasonal pattern was observed in *f*_RH_. Both *R*_S_ and *R*_H_ were sensitive to soil water content, with a reduction in respiration rate observed below 0.2 m^3^ m^−3^ soil water content. This was particularly evident on 5 March 2010 and 12 December 2010, the driest measurement dates (Fig. 2).

### Effects of warming and nitrogen addition on R_S_

On average, *R*_S_ was increased by 41% due to warming (p<0.0001) and by 12% due to nitrogen addition (p = 0.004). The treatments combined additively, as no significant interaction was observed. Warming significantly increased *R*_H_ by 37% (p = 0.014), though nitrogen did not significantly affect *R*_H_ (p = 0.798), nor was there a significant interaction between the warming and nitrogen treatments. The proportional contribution of *R*_H_ to *R*_S_ was reduced to an average of 59% by nitrogen addition, although this reduction was of marginal significance (p = 0.051, Table S4) due to high variability in *f*_RH_. Warming had no significant impact on *f*_RH_.

Responses of *R*_S_ and *R*_H_ to added substrate were highly variable across replicates, with values of *S*_I_ ranging from a 0.8 to 4.75 fold increase in respiration. There was no effect of the presence of roots on *S*_I_ (p = 0.487). For *R*_S_, maximum S_I_ was decreased significantly by warming (p = 0.040, Table 1). In contrast, nitrogen addition did not affect *S*_I_ significantly (p = 0.146), nor was there any significant interaction with warming (p = 0.835).

**Table 1:** Mean ± SE of soil carbon concentration, soil nitrogen concentration, microbial biomass, plant available nitrogen and substrate induced respiration (*S*_I_) by treatment.

	Treatment			
Variable	Control	Warming	Nitrogen	Warming ×N
Total carbon (g kg^−1^)	41.7±2.4	43.2±0.3	44.9±1.0	44.1±0.7
Total nitrogen (g kg^−1^)	3.3±0.2	3.4±0.1	3.5±0.1	3.5±0.1
Microbial biomass carbon (mg kg^−1^)	639±34	741±47	655±24	**604±25**
Plant-available nitrogen (mg m^−2^)	7.9±1.8	3.8±0.5	11.8±3.8	10.0±3.9
Root Biomass (g m^−2^)	389±51	454±27	518±83	482±30
*S*_I_	1.91±0.38	**1.27±0.14**	1.46±0.28	0.93±0.12

Bold indicates a significance level of: *p*≤0.05.

### Modelled temperature responses of R_S_

The presence of roots increased *R*_10_ significantly (p<0.0001, Table S5), but had no effect on *E*_0_ (p = 0.340). As both *R*_S_ and *R*_H_ were shown to be sensitive to soil water content, addition of the soil water content response function in Equation (1) resulted in a significant improvement in model fit over a temperature-only model (ΔAIC  = 1350, p<0.0001). The best-fit model of the temperature response of *R*_S_ included warming and nitrogen addition as fixed effects on *R*_10_, which was increased significantly by both warming (p<0.0001, Table S6) and nitrogen addition (p<0.0001, Table 2). *E*_0_ was unaffected by either warming or nitrogen and there were no significant interactions between warming and nitrogen for either *R*_10_ or *E*_0_, so they were removed from the model. The inclusion of a warming by measurement year interaction for *R*_10_ resulted in small (0.02 μmol m^−2^ s^−1^) and marginally significant decrease in *R*_10_ during the second year of warming (p = 0.065, Table S7). Inclusion of the temperature by year interaction for *E*_0_ resulted in a non-significant interaction term (p = 0.347), indicating little effect of treatment time on the temperature response of *R*_S_. For *R*_H_, all treatments exhibited a single temperature response curve regardless of treatment (Table 2).

**Table 2:** Mean ± SE parameter values for the temperature response of soil respiration, *R*_S_, and heterotrophic soil respiration, *R*_H_, generated by fitting Equation (1) to measured data using a nonlinear mixed-effects models; parameters supplied represent significant fixed effects in the final model.

Treatment		*R*_10_ (μmol m^−2^ s^−1^)	*E*_0_ (kJ mol^−1^)	*a*	*b*
*R*_S_	Control	0.77±0.03	326±6	0.62±0.05	12.36±0.79
	Warming	0.88±0.04	-	-	-
	Nitrogen	0.93±0.04	-	-	-
	Warming ×N	1.05±0.04	-	-	-
*R*_H_	Control	0.56±0.03	331±12	0.62±0.05†	12.36±0.79†

† fixed value, not fitted in the model.

### Variation of soil properties and microbial biomass

The average soil carbon concentration was 43 g kg^−1^ and this was not affected significantly by either warming or nitrogen addition (Table 1). Likewise, total nitrogen concentration, which averaged 3.4 g kg^−1^, was unaffected by any of the treatments. The average microbial biomass carbon was 646 mg kg^−1^. This did not change significantly under the main effects of warming and nitrogen addition. However, a significant negative interaction between warming and nitrogen addition was observed on the final soil sampling date (p = 0.023) indicating a reduced microbial biomass under combined warming and nitrogen addition. Cumulative exchange of plant available nitrogen, as estimated from the one month burial of PRS probes, was 8 mg N m^−2^ on average and was not significantly different between treatments. At the time of the final soil sample, average (± SE) root biomass in the top 100 mm of soil was 465±26 g m^−2^. Root biomass was highly variable and no treatment differences were detected.

## Discussion

The average (± SE) soil respiration rate measured in the control plots over the course of this study was 0.96±0.09 μmol m^−2^ s^−1^. This value falls well within the range reported for temperate grasslands [43]. The relative contribution of *R*_H_ to *R*_S_ of 71% for control plots was very close to the average for non-forest ecosystems of 63% [20] and also agreed well with another temperate grassland warming experiment where *R*_H_ contributed 56–72% of *R*_S_ annually [25]. The 3°C warming treatment led to an increased rate of both *R*_S_ and *R*_H_ over the 27 month measurement period. The average 41% increase in *R*_S_ due to warming falls well within the reported range of a 25% reduction to a 45% increase in *R*_S_, as a result of experimental warming [6]. Likewise, warming-induced enhancement of *R*_H_ has been observed in other grassland warming experiments [25]. Warming treatment had no significant impact on *f*_RH_ indicating that *R*_A_ and *R*_H_ were similarly sensitive to temperature. This is supported by the results of the temperature response curve fitting, in which similar values of *E*_0_ were obtained for both *R*_S_ and *R*_H_. However, the temperature response analysis did reveal a slightly higher basal respiration rate, *R*_10_, for warmed soils. As this increase in *R*_10_ was only evident in *R*_S_, and not *R*_H_, we must assume that *R*_A_ is responsible for the increase in *R*_10_. While an increase in the basal rate of *R*_A_ as a result of warming would appear to contradict the previous finding that *f*_RH_ was unaffected by warming, measurements of *f*_RH_ were highly variable and, as they were calculated from plot averages, subject to within plot temperature variation. Thus, our results are consistent with a slight increase in root activity in the warmed plots. As no differences in root biomass were observed as a result of warming, this increase in *R*_A_ may be due to increased specific root respiration. Further, long-term warming has been associated with increased root exudation [44], and labile carbon in these exudates may have stimulated rhizosphere microbial activity leading to an increase in *R*_A_, as measured by the root exclusion approach.

The acclimation of *R*_S_ to warming frequently observed in many long-running soil warming experiments [7], [8] was absent in our study. We expected that acclimation would result in a significant, negative warming by measurement year interaction (i.e., a decrease in *R*_10_ or *E*_0_ relative to the control during the second year of warming). However, we observed only a small, marginally significant treatment by measurement year interaction effect for *R*_10_. One possible explanation for this lack of acclimation is the relatively short duration of this experiment. Physiological acclimation of roots and soil microbes should occur rapidly compared with the duration of our experiment, though changes to biomass and soil carbon pools may take longer. Another explanation is that acclimation has been linked to depletion of labile carbon substrates [9], [10]. As the study site was recently cleared of vegetation and soil structure was disturbed, it is likely that the size of the labile carbon pool was reduced as a result of the disturbance. Our measurements would then reflect the temperature response of decomposition of more recalcitrant soil organic matter in the absence of a large labile carbon pool to which size adjustments can occur. As the system advances and labile carbon accumulates, acclimation may become evident. However, *S*_I_ was significantly higher in the control treatment, indicating that labile substrates represent a greater limitation to *R*_S_ in the control plots. This is consistent with observations of other grassland warming experiments which showed higher labile carbon content in warmed soils due to greater belowground allocation and turnover of roots [11].

The significant reduction in soil volumetric water content as a result of soil warming has potential implications for the effects of soil warming on *R*_S_. Both *R*_S_ and *R*_H_ were observed to be water limited below a soil volumetric water content value of 0.2 m^3^ m^−3^. Thus, warming-induced soil drying may serve to mitigate warming-enhanced carbon losses to *R*_S_, as other warming experiments have shown [45]. However, the soil-drying effects observed here were small except when water was already limiting in all treatments. We suggest that soil drying effects of warming are contributing little to the mitigation of warming effects at this site, due to the frequency of rainfall and the relatively short duration of water limited periods.

The 12% increase in *R*_S_ with nitrogen addition is consistent with findings for temperate grasslands [46]. In young and severely nitrogen limited ecosystems, added nitrogen may increase the amount of photosynthate allocated belowground [16]. This may be a reasonable explanation for increased *R*_S_ in our experimental tussock grassland, which was planted shortly before measurements began and has very low levels of plant-available nitrogen.

The increase in *R*_S_ due to nitrogen addition can be attributed entirely to *R*_A_, as *R*_H_ remained unaffected by nitrogen addition. The analysis of *f*_RH_ confirmed that autotrophic contribution to *R*_S_ increased with addition of nitrogen. Likewise, nitrogen increased *R*_10_ for *R*_S_, but had no effect on *R*_H_. Similar to warming, we found no significant increase in root biomass in the treatment with added nitrogen, though there was a trend for higher root biomass. Specific root respiration has been shown to increase with increasing root tissue nitrogen concentration in grasslands [47]. Thus, it is likely that increased specific root respiration rate as a result of nitrogen addition contributed to this increase in *R*_S_.

Contrary to expectations, plant-available nitrogen in the soil was not increased by warming or nitrogen addition. There may be several factors contributing to this result. First, nitrogen was applied to both the plant and soil. As a result, a portion of the nitrogen was likely intercepted by foliar uptake [48]. Additionally, the tussock grassland soils are subject to heavy leaching, likely decreasing the residence time of added nitrogen in soils. Further, the PRS probes used to estimate plant available nitrogen were inserted into soil with roots. Strong competition for nitrogen amongst roots may have contributed to the low level of plant-available nitrogen in all treatments.

No interactive effects of warming and nitrogen addition were observed for *R*_S_, *R*_H_ or their respective temperature responses. This indicates that the effects of these separate global change drivers are additive. It has been suggested that global change drivers may interact, resulting in smaller effect sizes than those reported for single drivers [28]. However, few such instances have been noted for *R*_S_ [49], [50]. The only significant interaction observed in this study was the negative interaction between warming and nitrogen addition on microbial biomass. Nitrogen addition generally results in decreased microbial biomass [26]. While we found no significant decrease of microbial biomass under nitrogen addition alone, a decrease was observed under combined warming and nitrogen addition. However, this decrease did not result in reduced *R*_H_.

Absent from this study is the inclusion of the rhizosphere priming effect in our estimate of *R*_H_. This refers to the effect that living roots have on *R*_H_ as a result of their impact on the physical and chemical environment within the soil [51]. Priming effects can influence both the rate and temperature response of *R*_H_ [52]–[54]. This may represent a potential source of error in our determination of the contribution of *R*_H_ to *R*_S_. A previous study in tussock grassland soils showed a dampening of the short-term response of *R*_H_ to temperature when plants were present [54]. However, in that study, priming effects were absent when plant and soil were held at a constant temperature of 15°C. Only when the soil temperature was perturbed from the constant incubation temperature over a period of hours were priming effects observed. As such, use of the root exclusion method may be appropriate for evaluating longer-term, seasonal temperature responses of *R*_H_, as in the present study.

**Figure 3:**
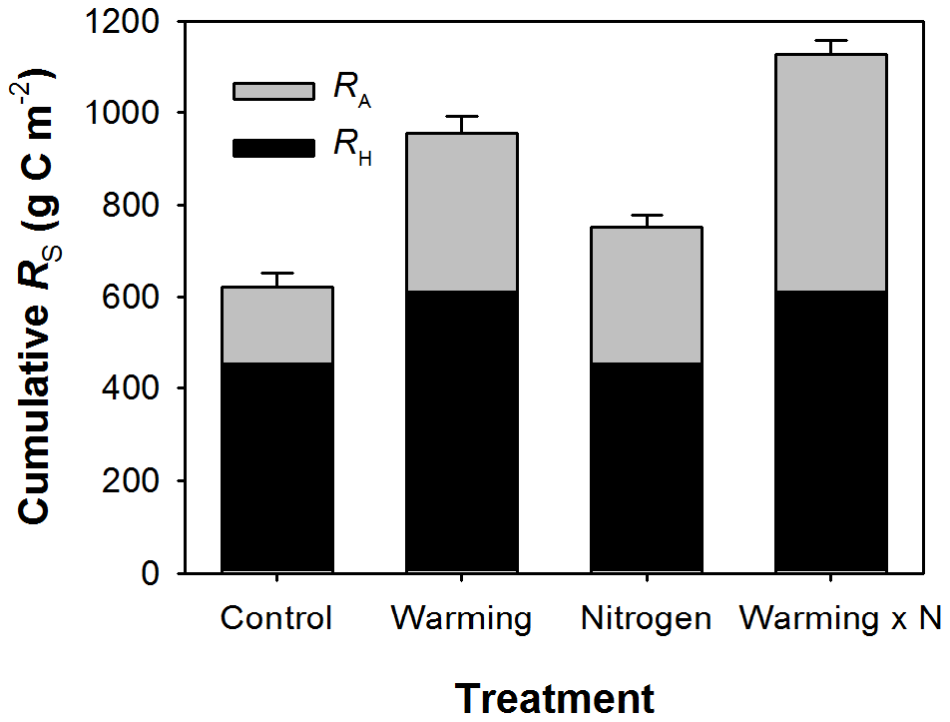
Cumulative soil respiration. Cumulative soil respiration, *R*_S_, for the entire 27 month study period partitioned between autotrophic, *R*_A_, and heterotrophic respiration, *R*_H_. Cumulative estimates were obtained from Equation (1) and parameter values from Table 2. Error bars were estimated from the 95% confidence interval of the linear relationship between measured and modelled *R*_S_.

Our results highlight the potential impacts of warming and nitrogen addition on the global carbon cycle. Over the course of the 27 month experiment, simulated cumulative CO_2_ emissions, based on measured temperature response curves of *R*_S_, were 621 g C m^−2^ for the control treatment (Fig. 3). Warming increased cumulative *R*_S_ to 953 g C m^−2^, nitrogen addition resulted in cumulative emissions of 750 g C m^−2^ and the combined effect resulted in emissions from *R*_S_ of 1127 g C m^−2^. While these represent substantial differences in emissions, the contrasting responses of autotrophic and heterotrophic respiration to the treatments must be considered. While increases in *R*_A_ may have consequences for the carbon economy of plants, they are likely to be offset by increased primary production. However, increases in *R*_H_ due to warming present the potential for sustained loss of stored soil carbon. Extrapolation of our results to the 4.3 Mha of tussock grassland in New Zealand suggests the additional 70 g C m^−2^ y^−1^ carbon losses to *R*_H_ as a result of 3 °C warming would amount to a positive feedback to rising atmospheric CO_2_ concentration equivalent to 30% of New Zealand's current annual fossil fuel emissions.

## Supporting Information

Table S1 **F-values for fixed effects in the best-fit linear mixed-effects model of soil volumetric water content.**
(DOC)

Table S2 **F-values for fixed effects in the best-fit linear mixed-effects model of soil respiration.**
(DOC)

Table S3 **F-values for fixed effects in the best-fit linear mixed-effects model of heterotrophic respiration.**
(DOC)

Table S4 **F-values for fixed effects in the best-fit linear mixed-effects model of the proportional contribution of heterotrophic respiration to total soil respiration.**
(DOC)

Table S5 **F-values for fixed effects in a nonlinear mixed-effects model of soil respiration including the effect of roots on** ***R*** **_10_ and** ***E*** **_0_ parameters.**
(DOC)

Table S6 **F-values for fixed effects in the best-fit nonlinear mixed-effects model of soil respiration.**
(DOC)

Table S7 **F-values for fixed effects in a nonlinear mixed-effects model of soil respiration including the interaction between the warming treatment and measurement year as a fixed effect on** ***R*** **_10_ and** ***E*** **_0_ parameters.**
(DOC)

Appendix S1 **Experimental layout for the Cass Warming Experiment.**
(DOC)
