# Supplementary material for: Effects of Soil Warming and Nitrogen Addition on Soil Respiration in a New Zealand Tussock Grassland
Source: PLoS One. 2014 Mar 12;9(3):e91204. doi: 10.1371/journal.pone.0091204 (PMC3951317; doi:10.1371/journal.pone.0091204)
Supplement: Table S1 — F-values for fixed effects in the best-fit linear mixed-effects model of soil volumetric water content. (DOC) [file pone.0091204.s001.doc]

**Table S1:** F-values for fixed effects in the best-fit linear mixed-effects model of soil volumetric water content, *θ*; numDF and denDF = numerator and denominator degrees of freedom.

|  | **numDF** | **denDF** | **F-value** | **p-value** |
| --- | --- | --- | --- | --- |
| (Intercept) | 1 | 3779 | 104358.4 | <0.0001 |
| Warming | 1 | 14 | 14.68 | 0.0018 |
| Nitrogen | 1 | 14 | 0.66 | 0.4301 |
| Date | 29 | 3779 | 704.87 | <0.0001 |
| Warming:Nitrogen | 1 | 14 | 0.22 | 0.6433 |
| Warming:Date | 29 | 3779 | 13.92 | <0.0001 |
| Nitrogen:Date | 29 | 3779 | 4.49 | <0.0001 |
| Warming:N:Date | 29 | 3779 | 2.05 | 0.0008 |

Fixed effects structure: *θ~*Warming*Nitrogen*Date; random effects: ~1|Plot/Collar
