# Supplementary material for: Effects of Soil Warming and Nitrogen Addition on Soil Respiration in a New Zealand Tussock Grassland
Source: PLoS One. 2014 Mar 12;9(3):e91204. doi: 10.1371/journal.pone.0091204 (PMC3951317; doi:10.1371/journal.pone.0091204)
Supplement: Table S2 — F-values for fixed effects in the best-fit linear mixed-effects model of soil respiration. (DOC) [file pone.0091204.s002.doc]

**Table S2:** F-values for fixed effects in the best-fit linear mixed-effects model of soil respiration, *R*_S_; numDF and denDF = numerator and denominator degrees of freedom.

|  | **numDF** | **denDF** | **F-value** | **p-value** |
| --- | --- | --- | --- | --- |
| (Intercept) | 1 | 2990 | 1267.774 | <0.0001 |
| Date | 29 | 2990 | 590.5977 | <0.0001 |
| Warming | 1 | 15 | 67.7692 | <0.0001 |
| Nitrogen | 1 | 15 | 11.4228 | 0.0041 |
| Date:Warming | 29 | 2990 | 5.5 | <0.0001 |
| Date:Nitrogen | 29 | 2990 | 3.9496 | <0.0001 |

Fixed effects structure: log(*R*_S_)~Warming*Date+Nitrogen*Date; random effects: ~1|Plot/Collar
