# Supplementary material for: Effects of Soil Warming and Nitrogen Addition on Soil Respiration in a New Zealand Tussock Grassland
Source: PLoS One. 2014 Mar 12;9(3):e91204. doi: 10.1371/journal.pone.0091204 (PMC3951317; doi:10.1371/journal.pone.0091204)
Supplement: Table S3 — F-values for fixed effects in the best-fit linear mixed-effects model of heterotrophic respiration. (DOC) [file pone.0091204.s003.doc]

**Table S3:** F-values for fixed effects in the best-fit linear mixed-effects model of heterotrophic respiration, *R*_H_; numDF and denDF = numerator and denominator degrees of freedom.

|  | **numDF** | **denDF** | **F-value** | **p-value** |
| --- | --- | --- | --- | --- |
| (Intercept) | 1 | 749 | 25.824 | <0.0001 |
| Date | 23 | 749 | 173.8512 | <0.0001 |
| Warming | 1 | 15 | 7.70911 | 0.0141 |
| Nitrogen | 1 | 15 | 0.0678 | 0.7981 |
| Date:Warming | 23 | 749 | 2.4632 | 0.0002 |
| Date:Nitrogen | 23 | 749 | 2.27274 | 0.0006 |

Fixed effects structure: log(*R*_H_)~Warming*Date+Nitrogen*Date; random effects: ~1|Plot/Collar
