# Supplementary material for: Effects of Soil Warming and Nitrogen Addition on Soil Respiration in a New Zealand Tussock Grassland
Source: PLoS One. 2014 Mar 12;9(3):e91204. doi: 10.1371/journal.pone.0091204 (PMC3951317; doi:10.1371/journal.pone.0091204)
Supplement: Table S4 — F-values for fixed effects in the best-fit linear mixed-effects model of the proportional contribution of heterotrophic respiration to total soil respiration. (DOC) [file pone.0091204.s004.doc]

**Table S4:** F-values for fixed effects in the best-fit linear mixed-effects model of the proportional contribution of heterotrophic respiration to total soil respiration, *f*_RH_; numDF and denDF = numerator and denominator degrees of freedom.

|  | **numDF** | **denDF** | **F-value** | **p-value** |
| --- | --- | --- | --- | --- |
| (Intercept) | 1 | 383 | 635.6696 | <0.0001 |
| Nitrogen | 1 | 16 | 4.4506 | 0.051 |
| Date | 23 | 383 | 8.9663 | <0.0001 |

Fixed effects structure: *f*_RH_~Nitrogen+Date; random effects: ~1|Plot
