# Supplementary material for: Effects of Soil Warming and Nitrogen Addition on Soil Respiration in a New Zealand Tussock Grassland
Source: PLoS One. 2014 Mar 12;9(3):e91204. doi: 10.1371/journal.pone.0091204 (PMC3951317; doi:10.1371/journal.pone.0091204)
Supplement: Table S5 — F-values for fixed effects in a nonlinear mixed-effects model of soil respiration including the effect of roots on R 10 and E 0 parameters. (DOC) [file pone.0091204.s005.doc]

**Table S5:** F-values for fixed effects in a nonlinear mixed-effects model of soil respiration, *R*_S_, including the effect of roots on *R*_10_ and *E*_0_ parameters; numDF and denDF = numerator and denominator degrees of freedom.

|  | **numDF** | **denDF** | **F-value** | **p-value** |
| --- | --- | --- | --- | --- |
| *R*10.(Intercept) | 1 | 3890 | 2040.909 | <0.0001 |
| *R*10.Roots | 1 | 3890 | 248.0078 | <0.0001 |
| *E*0.(Intercept) | 1 | 3890 | 3090.654 | <0.0001 |
| *E*0.Roots | 1 | 3890 | 0.9089 | 0.3405 |
| *a* | 1 | 3890 | 0.2552 | 0.6134 |
| *b* | 1 | 3890 | 263.2897 | <0.0001 |

Fixed effects structure: *R*_10_+*E*_0_~Roots, *a*+*b*~1; random effects: *R*_10_+*E*_0_~1|Plot/Collar
