# Supplementary material for: Effects of Soil Warming and Nitrogen Addition on Soil Respiration in a New Zealand Tussock Grassland
Source: PLoS One. 2014 Mar 12;9(3):e91204. doi: 10.1371/journal.pone.0091204 (PMC3951317; doi:10.1371/journal.pone.0091204)
Supplement: Table S6 — F-values for fixed effects in the best-fit nonlinear mixed-effects model of soil respiration. (DOC) [file pone.0091204.s006.doc]

**Table S6:** F-values for fixed effects in the best-fit nonlinear mixed-effects model of soil respiration, *R*_S_; numDF and denDF = numerator and denominator degrees of freedom.

|  | **numDF** | **denDF** | **F-value** | **p-value** |
| --- | --- | --- | --- | --- |
| *R*_10_.(Intercept) | 1 | 3072 | 2997.032 | <0.0001 |
| *R*_10_.Warming | 1 | 3072 | 16.133 | 0.0001 |
| *R*_10_.Nitrogen | 1 | 3072 | 17.76 | <0.0001 |
| *E*_0_ | 1 | 3072 | 3567.4 | <0.0001 |
| *a* | 1 | 3072 | 2.616 | 0.1059 |
| *b* | 1 | 3072 | 241.433 | <0.0001 |

Fixed effects structure: *R*_10_ ~Warming+Nitrogen, *E*_0_+ *a*+*b*~1; random effects: *R*_10_+*E*_0_~1|Plot/Collar
