# Supplementary material for: Effects of Soil Warming and Nitrogen Addition on Soil Respiration in a New Zealand Tussock Grassland
Source: PLoS One. 2014 Mar 12;9(3):e91204. doi: 10.1371/journal.pone.0091204 (PMC3951317; doi:10.1371/journal.pone.0091204)
Supplement: Table S7 — F-values for fixed effects in a nonlinear mixed-effects model of soil respiration including the interaction between the warming treatment and measurement year as a fixed effect on R 10 and E 0 parameters. (DOC) [file pone.0091204.s007.doc]

**Table S7:** F-values for fixed effects in a nonlinear mixed-effects model of soil respiration, *R*_S_, including the interaction between the warming treatment and measurement year as a fixed effect on *R*_10_ and *E*_0_ parameters; numDF and denDF = numerator and denominator degrees of freedom.

|  | **numDF** | **denDF** | **F-value** | **p-value** |
| --- | --- | --- | --- | --- |
| *R*_10_.(Intercept) | 1 | 3067 | 3199.196 | <0.0001 |
| *R*_10_.Warming | 1 | 3067 | 15.995 | 0.0001 |
| *R*_10_.Year | 1 | 3067 | 113.88 | <0.0001 |
| *R*_10_.Nitrogen | 1 | 3067 | 17.42 | <0.0001 |
| *R*_10_.Warming:Year | 1 | 3067 | 3.396 | 0.0654 |
| *E*_0_.(Intercept) | 1 | 3067 | 3901.88 | <0.0001 |
| *E*_0_.Warming | 1 | 3067 | 0.28 | 0.5968 |
| *E*_0_.Year | 1 | 3067 | 9.656 | 0.0019 |
| *E*_0_.Warming:Year | 1 | 3067 | 0.884 | 0.3471 |
| *a* | 1 | 3067 | 0.095 | 0.758 |
| *b* | 1 | 3067 | 273.13 | <0.0001 |

Fixed effects structure: *R*_10_ ~Warming*Year+Nitrogen, *E*_0_~Warming*Year, *a*+*b*~1; random effects: *R*_10_+*E*_0_~1|Plot/Collar
