# Supplementary material for: Effects of Soil Warming and Nitrogen Addition on Soil Respiration in a New Zealand Tussock Grassland
Source: PLoS One. 2014 Mar 12;9(3):e91204. doi: 10.1371/journal.pone.0091204 (PMC3951317; doi:10.1371/journal.pone.0091204)
Supplement: Appendix S1 — Experimental layout for the Cass Warming Experiment. (DOC) [file pone.0091204.s008.doc]

**Appendix S1: Experimental layout for the Cass Warming Experiment.**


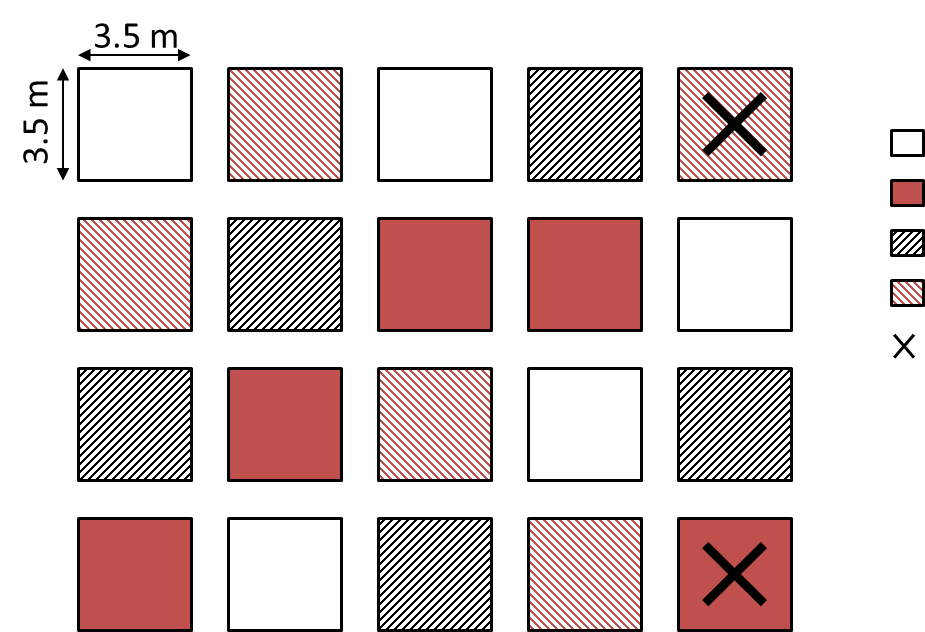


Control

Warming

Nitrogen addition

Warming and nitrogen

Cable malfunction

**Figure S1:** Diagram illustrating plot and treatment layout of the Cass Warming Experiment.


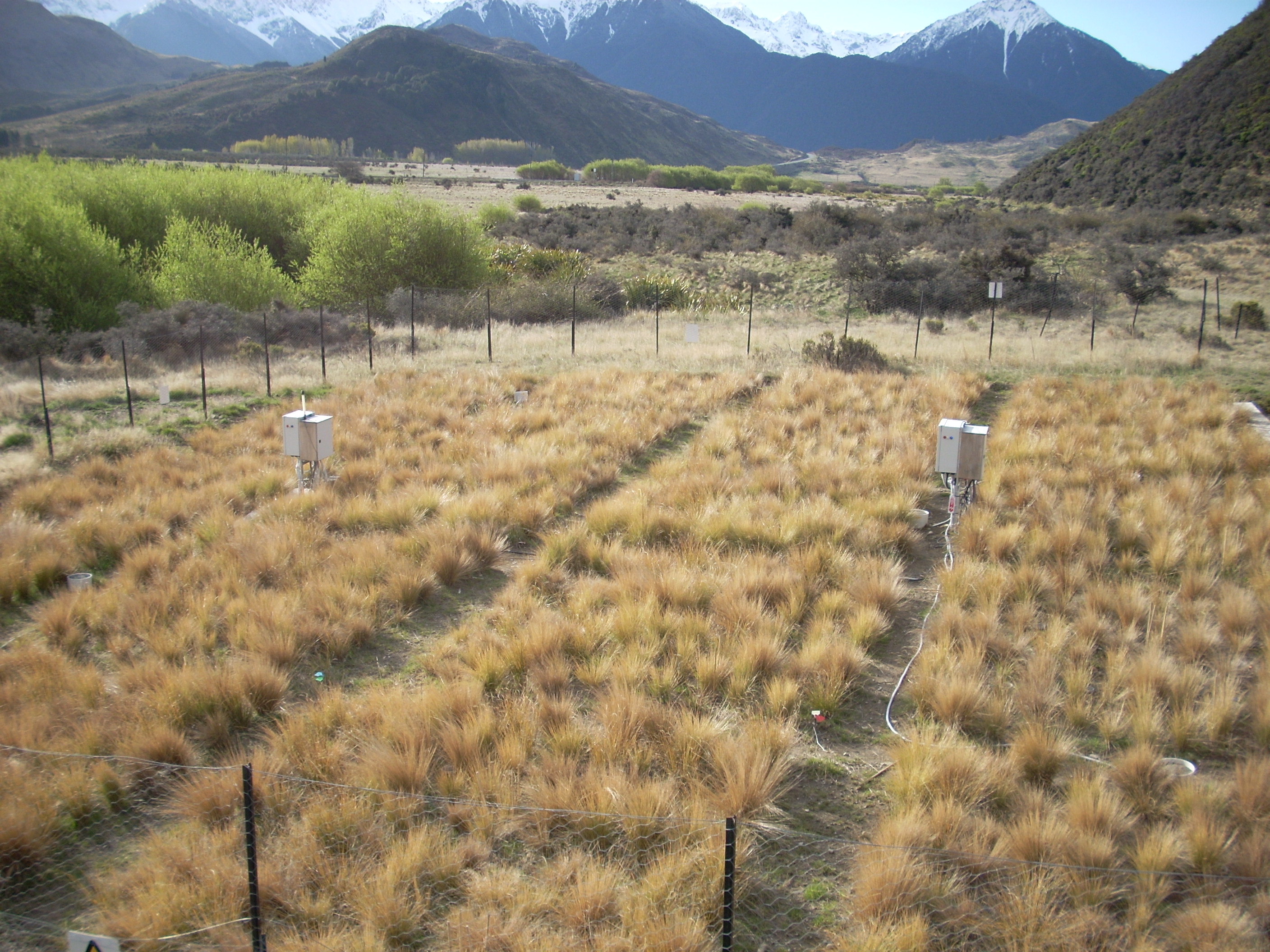

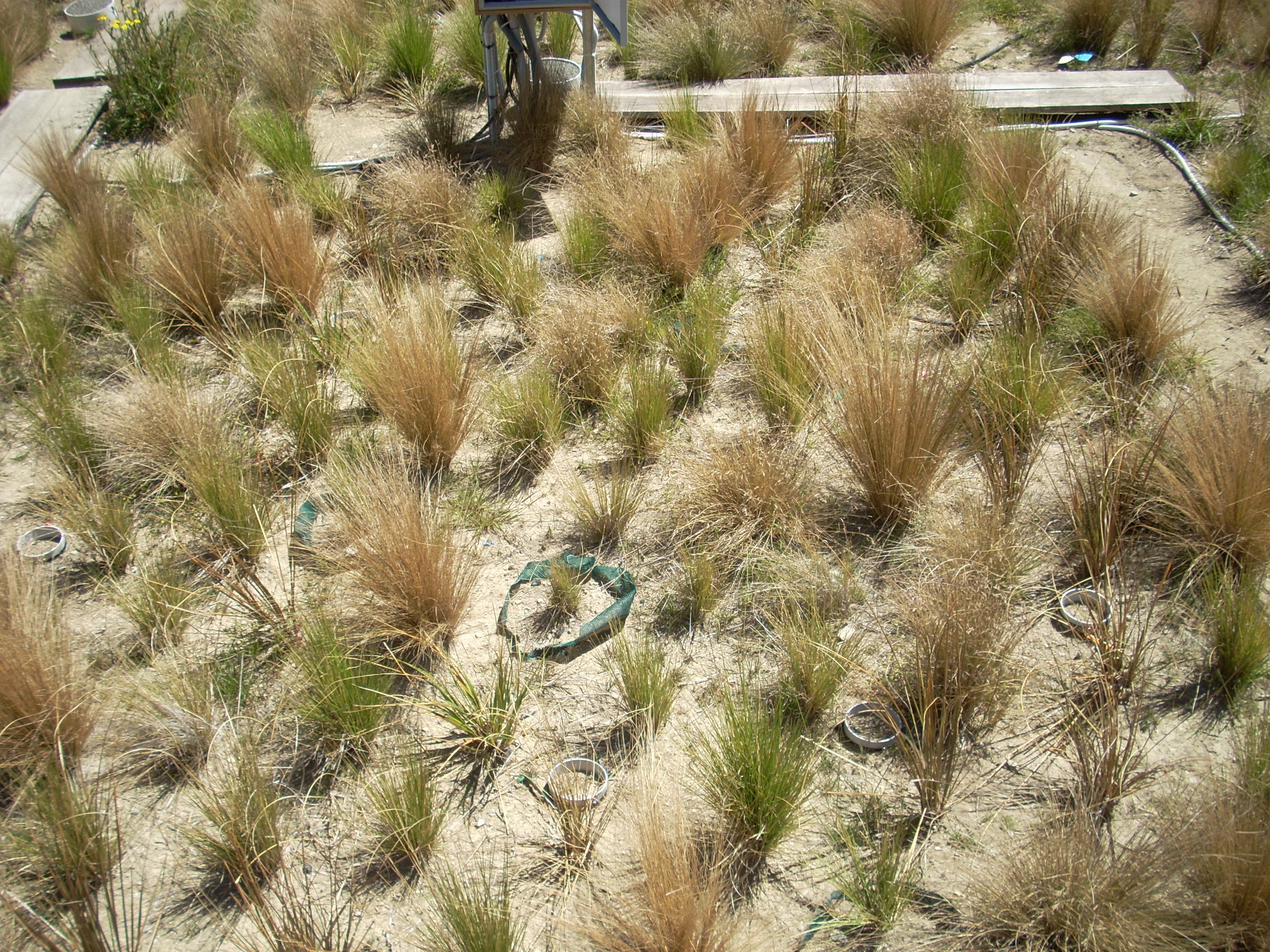


A

B

**Figure S2:** Photo of (A) the Cass Warming Experiment and (B) an example plot showing soil respiration measurement collars.
